# An updated overview of HPV-associated head and neck carcinomas

**DOI:** 10.18632/oncotarget.1934

**Published:** 2014-05-01

**Authors:** Apostolos Zaravinos

**Affiliations:** ^1^ Molecular Medicine Research Center and Laboratory of Molecular and Medical Genetics, Department of Biological Sciences, University of Cyprus, 1678 Nicosia, Cyprus; ^2^ Division of Clinical Immunology and Transfusion Medicine, Department of Laboratory Medicine, Karolinska Institutet, 14145 Huddinge, Sweden

**Keywords:** Head and neck squamous-cell carcinoma, human papilloma virus, oropharyngeal squamous cell carcinoma, p16INK4A, PD-1, PDL-1, CTLA-4, HPV vaccines, therapeutic cancer vaccines, management of HPV-induced HNSCCs

## Abstract

Human papilloma virus (HPV)-associated head and neck carcinoma is quite heterogeneous and most of the tumors arise in the oral cavity, oropharynx, hypopharynx and larynx. HPV was just recently recognized as an emerging risk factor for oropharyngeal squamous cell carcinoma (OSCC). HPV(+) tumors represent 5-20% of all head and neck squamous-cell carcinomas (HNSCCs) and 40-90% of those arising from the oropharynx, with widely variable rates depending on the geographic area, population, relative prevalence of environment-related SCC and detection assay. Different carcinogenic mechanisms are most likely implicated in cervical and oropharyngeal carcinogenesis. The most certain carcinogenic genotype for the head and neck region and the most common high-risk HPV genotype, HPV-16, is frequently detected in OSCC. A combination of p16^INK4A^ expression and molecular detection of HPV DNA is the gold standard for the viral identification in tissue and exfoliated cell samples. Differences in the biology of HPV(+) and HPV(-) OSCC may have implications for the management of patients. New immunotherapy drugs based on the release of the co-inhibitory receptors, cytotoxic T-lymphocyte-associated antigen 4 (CTLA-4) and programmed-death 1 (PD-1) have currently emerged. The goal of therapeutic cancer vaccination is inculcation of a persistent, tumor antigen-specific T cell response which kills tumor cells. The efficacy of the current HPV vaccines, Cervarix and Gardasil, in preventing HPV-related HNSCC is at present unknown. Treatment de-escalation is recommended as the current management of HPV-induced HNSCCs.

## Human papillomaviruses (HPVs)

Human papillomaviruses (HPVs) are small double-stranded DNA viruses that comprise a heterogeneous family consisting of more than 130 different HPV types [1]. Different HPV types have been detected in the anogenital tract, urethra, skin, larynx, tracheo-bronchial and oral mucosa and can cause a wide range of infections, including common warts, genital warts, recurrent respiratory papillomatosis, low-grade and high-grade squamous intraepithelial lesions (SILs), anal cancer, vaginal cancer and cervical cancer. Based on their association with cervical cancer, HPV types are classified as high-risk (HPV-16, 18, 31, 33, 35, 39, 45, 51, 52, 56, 58, 59, 68, 73 and 82) or low-risk (HPV-26, 30, 34, 53, 66, 67, 69, 70, 73, 82, 85) [2]. Evidence of the potential role of HPV in other tumor types has been shown, as well [3-8]. High-risk HPV types contribute significantly to viral associated neoplasms, accounting for approximately 600,000 cases (5%) of cancers worldwide annually [9]. In particular, HPV-16 accounts for approximately 50% of cervical carcinomas and more than 90% of HPV(+) carcinomas of the oropharynx (and the other ano-genital sites). Low-risk HPVs have been associated with benign warts of oral and urogenital epithelium in adults as well as children and they are only rarely found in malignant tumors. HPV has also been associated with several other types of SCC and their precursors at different sites, such as skin, vulva, vagina, penis, esophagus, conjunctiva, paranasal sinuses, and bronchus; but its role in the pathogenesis of the lesions is less clear than in cervical cancer. The similarity of the morphologic features of genital and oral HPV associated lesions was one of the early findings that raised the possibility that HPV might be involved in oral and laryngeal SCCs [10, 11]. Until recently, however, the role of HPV in the pathogenesis of head and neck squamous-cell carcinoma (HNSCC) has been quite uncertain.

HPV infections are mainly sexually transmitted through direct skin or mucosa contact and represent the most common sexually transmitted infections worldwide. The probability of transmission is very high, with an estimated life-time risk of cervical HPV infection in sexually active women of up to 80%. Exposure to HPV is determined by well known risk factors for most sexually transmitted infections, while determinants of susceptibility and infectivity are much less established [12]. Knowledge on the natural history of HPV infection derives from studies on cervical infection. The large majority of infections clear spontaneously within 24 months, although the time depends on the HPV type and the immune status. Clearing an infection does not always create immunity if there is a new or continuing source of infection [13]. The virus can either be completely cleared or remain in a latent form which can be later reactivated. Only a small fraction of infections cause clinical lesions; spontaneous regression occurs in most low grade lesions and in a fraction of high grade ones, while progression to invasive cancer is a very rare event and is preventable by surgical treatment of high grade lesions [14].

## Epidemiological data of head and neck carcinomas and HPV involvement

Head and neck carcinomas (HNCs) is the sixth most common cancer with an annual incidence of ~400.000 cases [15] and represents about 3.5% of all malignant tumors in the western societies [16, 17] and other parts of the world. HNC is quite heterogeneous and most of the tumors arise in the oral cavity, oropharynx, hypopharynx and larynx. Nearly 90% of these cancers are HNSCCs. The disease is associated with a poor prognosis, with a 5-year survival rate less than 50%. The most well-established risk factors for HNSCC are tobacco and alcohol abuse [18].

HPV involvement in head and neck carcinogenesis was initially reported 30 years ago [10, 11]; however, it was just recently recognized as an emerging risk factor for oropharyngeal squamous cell carcinoma (OSCC) [19]. OSCC begins in the oropharynx, the middle part of the throat that includes the soft palate, the base of the tongue, the tonsils and the side and back wall of the throat. Incidence of HPV(+) OSCC varies greatly worldwide from 25-80% and it is predicted to increase in the near future. OSCC now represents a significantly higher proportion of HNSCCs. This rise in incidence is mostly occurring in individuals aged 40-55 years, without environmental risk factors, and is associated with persistent infection with high-risk HPVs [20]. HPV(+) OSCC patients tend to be younger than HPV(-) ones [21]. Tonsil and oropharyngeal cancers increased in male predominance over the last 30 years, despite a decline in smoking, which may be linked to the decreasing proportion of HPV(-) cancers; while changes in sexual activity may be reflected in increasing proportion of HPV(+) cancers [20]. Recently, HPV-associated OSCC represents about 60% of OSCC cases compared to 40% in the previous decade [22]. In the USA, Sweden, Finland and Czech Republic an increasing incidence of OSCC has been observed during the last decade [23-26].

Nevertheless, the actual incidence of high-risk HPV infection in sites outside the oropharynx, as well as what is the best HPV detection method in HNSCC, have not yet been answered with confidence. Among the other extra-oropharyngeal subsites, HPV might have a role in the supraglottic larynx [27], whose marginal region is contiguous with the oropharynx, and it may account for the high-risk HPV infection rate reported in laryngeal SCCs [28, 29]. HPV detection rates were recently found to range between 12.6-90.9% in oropharyngeal carcinoma [30]. Only in one study the HPV detection rate was <20%; whereas in 34 other investigations it ranged between 20-40%. Also in 2 studies it ranged between 20-30% and in 15 studies HPV was detected in >40% of the tested samples. HPV detection rates, including high risk HPV viral load, were found to be significantly higher in tonsillar cancers than in other head and neck carcinomas [31, 32]. As for the oral cavity SCCs, many authors reported frequent high-risk HPV involvement by considering the over-expression of p16^INK4A^ as equivalent to HPV infection [33, 34]. Nevertheless, recent data in oral cancers indicate that p16^INK4A^ over-expression is due to different mechanisms and high-risk HPV infection is very rarely detectable in oral SCCs [27, 35].

Among the many high-risk HPV types, HPV-16 is the most common, found in almost 90% of the HPV(+) oropharyngeal cancers. At present, HPV-16 remains the only HPV type that is classified as cancer-causing in the head and neck [2, 36]. In addition, there is a more diverse spectrum of other high-risk HPV types with a less important role and a putatively different behavior than that of HPV-16 [37]. Of these, HPV-33, HPV-35, HPV-45 and HPV-58 have been detected in lower frequencies, representing 10-15% of HPV(+) OPC [38-41]. Therefore, HPV(+) OSCCs belong to a distinct clinical and molecular entity with a looser association with tobacco and alcohol.

## Molecular mechanisms through which HPVs induce carcinogenesis

The HPV genome is composed of six early (E1, E2, E4, E5, E6, and E7), two late (L1 and L2) open reading frames, and a non-coding long control region (LCR) [42]. E5, E6 and E7 genes encode three viral onco-proteins. E6/E7 proteins function as the dominant onco-proteins of high-risk HPVs inactivating the tumor suppressor proteins, p53 and pRb, respectively. E6 and E7 genes [43] can modify the cell cycle so as to retain the differentiating host keratinocyte in a state that is favorable to the amplification of viral genome replication and consequent late gene expression.

HPV E6 in association with host ubiquitin ligase E6-associated protein (E6AP) acts to ubiquitinate p53, leading to its proteasomal degradation [44]. P53 is a well-studied transcription factor that induces cell cycle arrest or apoptosis in response to cellular stress or DNA damage, and has been attributed the roles of “guardian of the genome” and “policeman of the oncogenes”. The first role consists in sensing and reacting to DNA damage through the ATM/ATR and Chk1/Chk2 kinases, and the second in responding to oncogenic signaling through the p53-stabilizing protein ARF [45].While in most cancers p53 malfunction is determined by p53 mutations, in HPV-associated carcinomas wild-type functional p53 is degraded by E6 oncoprotein. Moreover, cells expressing HPV-16 E6 show chromosomal instability [46, 47]. HPV E7 on the other hand inactivates pRb, which controls the G1-S phase transition of the cell cycle by binding the transcription factor E2F. As a consequence, E2F is released with consequent promotion of cell G1-S phase transition [48, 49] and transcription of genes, such as cyclin E and cyclin A, which are required for cell cycle progression. This functional inactivation of pRb results in a reciprocal over-expression of p16^INK4A^. The HPV(+) tonsillar SCC share a disruption of the pRb pathway as a common biological marker. By immunohistochemistry (IHC), most HPV(+) HNSCCs show p16^INK4A^ over-expression. In non-HPV-related HNSCC, continuous tobacco and alcohol exposure can lead to mutational loss of the p16^INK4A^ and p53 genes. These early neoplastic events are detected in 80% of HNSCCs and cause uncontrolled cellular growth [50]. The expression of p53 and bcl-2 is not associated with HPV(+) oral cavity SCC [51] and mutations in p53 are rarely seen in HPV(+) tumors compared with HPV(-) tumors [52]. Furthermore, there seems to be an inverse relationship between epidermal growth factor receptor (EGFR) expression and HPV status. For patients with OSCC, high p16^INK4A^ and low EGFR were associated with improved outcome, suggesting a predictive role in surgically treated patients [53]. All HPVs can induce transient proliferation, but only HPV-16 and HPV-18 can immortalize cell lines in vitro. Carcinogenic mechanisms in HPV-associated OSCCs may be similar to those in cervical cancers. However, since the oral cavity and the oropharynx are exposed to higher levels of chemical carcinogens compared to the genital tract, it is likely that different mechanisms are implicated in cervical and oropharyngeal carcinogenesis.

## HPV detection methods in OSCC

Although the management of OSCC does not require evaluation of HPV status, HPV-testing in OSCC patients is increasingly becoming the standard of care. HPV-induced OSCC constitutes a separate tumor entity with distinct clinical and histopathological features, improved performance status and better prognosis. Nevertheless, heterogeneity both in biological and clinical behavior among HPV(+) cases has been well observed [54]. This heterogeneity highlights the need to assess the presence of HPV in the tumor using an algorithm that can detect just the biologically active virus, and identify the cases with improved clinical outcome. Molecular detection of HPV DNA is the gold standard for the identification of HPV in tissue and exfoliated cell samples using several assays with different sensitivity and specificity, including Southern transfer hybridization, dot blot hybridization, in situ hybridization (ISH), hybrid capture and polymerase chain reaction (PCR) [55]. All the limitations and advantages of each method have been previously described in detail [55].

## p16^INK4A^ immunostaining in conjunction with HPV DNA detection is a useful tool to establish a diagnosis of HPV-related OSCC

HPV-related and HPV-unrelated OSCCs show different genetic signatures which most likely underlie differences in tumor development and progression [56]. These differences may also have implications for the management of patients [57]. The detection of elevated p16^INK4A^ protein levels by IHC is the most well-known biomarker for the detection of biologically active HPV infection in HNSCC [58]. p16^INK4A^ is a cyclin-dependent kinase (CDK) inhibitor, encoded by the CDKN2A locus, which arrests the cell cycle in the G1 stage [59, 60]. pRb inactivation by HPV E7 is associated with up-regulation of CDKN2A and consequent protein over-expression. Conversely, in HPV-unrelated, environment-related HNSCC, perturbation of the pRb-pathway is uncommon and CDKN2A expression is usually low. Therefore, p16^INK4A^ immunostaining in conjunction with HPV DNA detection is very a useful tool to establish a diagnosis of HPV-related OSCC [53]. Weinberger et al. [61] demonstrated that HPV(+) and p16^INK4A^(+) tumors had favorable prognosis and the presence of HPV in the tumors per se did not have a substantial positive impact on prognosis. As p16^INK4A^ expression lacks specificity for high-risk HPV and does not distinguish p16^INK4A^ up-regulation due to E7-mediated pRb loss from that sustained by other so far unidentified mechanisms (e.g., stress, aging, senescence, etc.), and given the different outcomes in the p16^INK4A^(+)/HPV(-) subgroups, in the context of personalized treatments, p16^INK4A^(+)/HPV(-) OSCCs should be considered as a distinct subset. For this reason, it is recommended that HPV should be assessed both by ISH and p16^INK4A^ [62].

In the Danish Head and Neck Cancer Group (DAHANCA) 5 trial [63] p16^INK4A^ was evaluated as prognostic marker of treatment response and survival in a cohort of patients treated solely with conventional radiotherapy. p16^INK4A^ positivity was detected in 22% of the tumors; however, no substantial difference was observed between p16^INK4A^(+) and p16^INK4A^(-) tumors. Specifically, p16^INK4A^(+) tumors seemed to be more closely associated with poor histopathologic differentiation compared with the p16^INK4A^(-) ones, but the difference was not statistically significant, indicating that p16^INK4A^ alone is not an adequate marker. The weakness of this study is that the authors included many p16^INK4A^(+) tumors that were not HPV(+) in the analysis as if they were HPV(+).

Preclinical data for HNSCC cell lines and xenografts showed more antitumor activity when treated with the anti-EGFR monoclonal antibody panitumumab combined with radiotherapy, than when treated with radiotherapy alone. Furthermore, phase 1 response data for panitumumab plus chemotherapy suggested that additional investigation of panitumumab in HNSCC is needed [64]. In the Study of Panitumamub Efficacy in Patients With Recurrent and/or Metastatic Head and Neck Cancer (SPECTRUM), panitumumab plus cisplatin and fluorouracil was compared with chemotherapy in patients with recurrent or metastatic HNSCC. Overall survival did not significantly improve with the addition of panitumumab to the chemotherapy regimen; however, improvements were recorded in progression-free survival and objective response. Furthermore, in a retrospective analysis, a negative HPV tumor status predicted overall and progression-free survival after treatment with cisplatin and fluorouracil plus panitumumab. Moreover, a p16^INK4A^(+) status was a favorable prognostic marker in patients who received only chemotherapy, suggesting a potential prognostic effect in this population of patients. The authors reported that the p16^INK4A^ status of the tumor, regional differences in overall survival, as well as other factors including the intensity and amount of previous treatment, might be important considerations in the design of future global trials in recurrent or metastatic HNSCC. However, the drawback of this study is that conclusions about EGFR inhibition were erroneously drawn based on the patients' p16^INK4A^ status, since half of the tumors were rated as HPV(+), just by p16^INK4A^(+) test.

The conclusion of these two studies is that presence of HPV DNA in tissue biopsies is not always sufficient to attribute a cancer of the oropharynx to HPV, depending on the different sensitivity of the various assays relying on DNA detection (especially in tobacco/alcohol exposed patients). Appropriate algorithms should be used to define an HPV-induced tumor. Assessment of HPV status is indicated in patients with oropharyngeal carcinomas, particularly when no environmental risk factors are present and in patients with neck metastasis and carcinoma of unknown primary as HPV detection in metastatic lymph node samples is strongly indicative of a primary in the tonsils or in the base of the tongue [65].

## Prognosis of HPV-induced carcinomas

The first line of evidence of the impact of HPV in prognosis comes from various small single-institutional retrospective case series, showing that patients with HPV(+) HNSCC (particularly those with oropharyngeal primary) treated by radiotherapy, chemoradiotherapy, surgery or combined modality therapy, have better outcome than those with HPV-uninduced cancer [66, 67]. HPV(+) SCC patients were estimated to have up to an 80% reduction in risk of disease failure compared to HPV(-) patients. Furthermore, retrospective analyses of archival tumor specimens from patients enrolled in phase II and III trials, which received more specific treatment regimens [68, 69] and meta-analyses [70, 71], confirmed that HPV(+) HNSCC is a separate biologic entity and that these patients have significantly better prognosis than patients with HPV-unrelated tumors. In these studies, the survival benefit was most predominant or restricted in patients with an oropharyngeal primary tumor. Furthermore, patients with HPV(+) HNSCCs, OSCCs and tonsillar SCCs have lower disease specific mortality and are less likely to experience progression or recurrence of their cancer than HPV(-) patients [72]. The reason why patients with HPV-induced HNSCC have better prognosis than those with HPV-unrelated cancer remains to be explained. Robust data indicate that cigarette smoking may modify the clinical behavior of HPV(+) SCC, adversely affecting the prognosis of these neoplasms [73]. Recently, a recursive partitioning analysis showed that the combination of tumor HPV status, smoking and TN category segregates patients with stage III and IV OSCCs into 3 groups with different prognoses: patients with HPV-induced SCCs were considered to be at low risk, with the exception of smokers with advanced nodal category, who were considered to be at intermediate risk; patients with HPV(-) SCCs were considered to be at high risk, with the exception of non-smokers with tumors of stage T2 or T3, who were considered to be at intermediate risk [74]. Some authors have argued that HPV status may reduce the overall prognostic significance of nodal category [75]. As mentioned above, the high-risk HPV E6 and E7 oncoproteins are prognostic factors in HNSCC. The E7-mediated inactivation of pRb is associated with CDKN2A/p16^INK4A^ up-regulation [76]. The absence of p53 mutations is significantly associated with better overall survival. Also, p16^INK4A^ positivity is associated with better outcomes, regardless of HPV positivity. As a consequence, the survival benefit observed in HPV-induced HNSCC may not be the result of HPV positivity per se, but rather the result of the absence of p53 gene mutations or p16^INK4A^ deletion in HPV(+) tumors, which are responsible for poor prognosis in HPV(-) patients [77]. Another unclear aspect is whether HPV status is a prognostic marker, a predictive marker for response to a specific treatment, or both. So far, the data support the hypothesis that HPV positivity results in a survival benefit, independently of treatment. However, large randomized clinical trials including the stratification of patients according to HPV status are needed to provide a definite response.

## New immunotherapy drugs anti-CTLA-4 and anti-PD-L1 block co-inhibitory signaling

**Figure 1:**
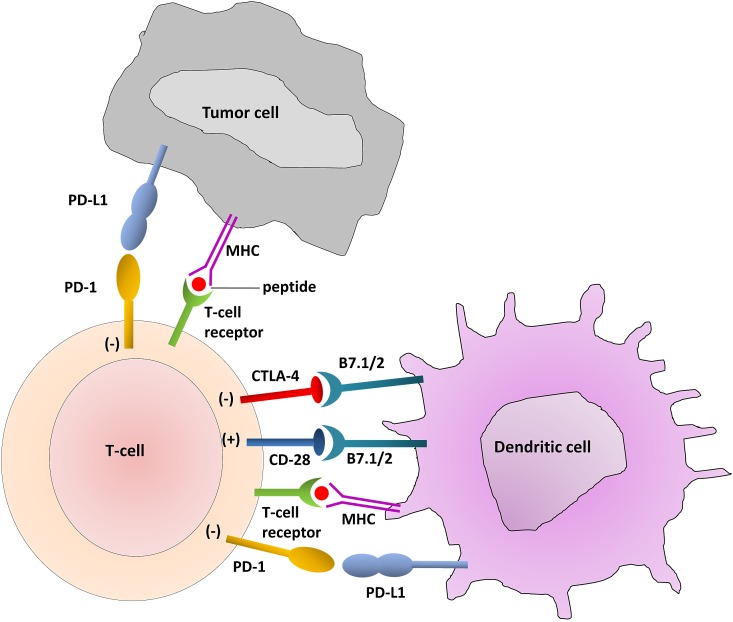
T-Cell Interaction with dendritic cells and tumor cells The immune checkpoints CTLA-4 and PD-1/PD-L1 are highlighted in the interactions among T-cells, dendritic cells and tumor cells.

Both in environmental-induced carcinogenesis and HPV oncogene-induced transformation, HNSCC is associated with a fundamental failure of immune surveillance, where tumor cells have escaped recognition and lysis by the cytotoxic T lymphocytes (CTLs) of adaptive immunity. The critical effector cells of adaptive antitumor immunity are the activated CD8(+) CTLs. Activation of the naïve, antigen-restricted CD8(+) CTLs first requires binding of the T cell receptor (TCR) to its cognate tumor antigen (TA) in complex with human leukocyte antigen (HLA) I. Although the engagement between a tumor antigen and a T cell receptor (TA-TCR engagement) is necessary, it is not sufficient for CTL activation and tumor cytolysis. Initial activation also depends upon the balance between co-stimulatory and co-inhibitory signaling by dendritic cells (DCs) and CD4(+) helper T cells, as well as freedom from suppression caused by CD4(+) regulatory T cells (Tregs). HNSCC elicits T cell anergy in both peripheral and tumor-infiltrating lymphocytes (TILs). Functional defects in TILs include low production and response to IL-2 [78]; vulnerability to spontaneous apoptosis, mediated by the Fas/Fas-ligand pathway [79]; low expression of CD3-f, OX40, and 4-1BB, co-stimulatory molecules required for signaling by the TCR [78, 80]; and high expression of co-inhibitory receptors, cytotoxic T-lymphocyte-associated antigen 4 (CTLA-4) and programmed-death 1 (PD-1) [78, 81] (Figure 1).

Successful activation of the CTL following the binding of TA-TCR depends upon predominance of co-stimulatory versus co-inhibitory signaling by accessory receptors. The co-inhibitory receptors CTLA-4 and PD-1 down-modulate CTL response in the setting of chronic antigen stimulation - a useful adaptation for resolving the inflammatory response following infection and in preventing auto-immunity. However, in the setting of cancer these receptors induce pathologic tolerance. A new therapeutic paradigm is the design of mAbs to block co-inhibitory signaling, releasing the CTL from anergy. The first in class is ipilimumab, an IgG1 mAb against the CTLA-4 co-inhibitory receptor expressed on activated CTLs and Tregs. CTLA-4 and the major co-stimulatory receptor CD28 compete for the same ligand on antigen-presenting cells (APCs), B7. The blockade of CTLA-4, releases B7 to bind CD28, thus propagating the B7-CD28 co-stimulatory signal that is required for TA-specific TCR activation. Ipilimumab has been FDA-approved in melanoma [82]. Release from CTLA-4 co-inhibitory signaling appears to up-regulate TA-specific CTLs and mediate therapeutic response. Blockade of constitutive CTLA-4 signaling in Tregs also potentiates response [83]. However, non-specific up-regulation of CTLs can lead to significant autoimmune adverse events [84]. A similar drug, the IgG2 mAb tremelimumab, is also under development [84]. CTLA-4 checkpoint inhibitors have striking potential to release T-cell immune-suppression in HNSCC, where the immune microenvironment is characterized by CTL anergy and Treg infiltration.

PD-1 is a second inhibitory member of the CD28/CTLA-4 family of co-receptors. It is expressed on CTLs, NK cells, B cells and macrophages, and it is thought to be a broader negative regulator of immune response than CTLA-4. PD-1 has two ligands: PD-L1 and PD-L2. PD-L1 is up-regulated on DCs and macrophages in response to chronic antigen stimulation, as is the case in the tumor microenvironment; many tumors including HNSCC co-opt expression of PDL1 to induce CTL and NK anergy. Therapeutic mAbs against both PD-1 and PD-L1 are entering advanced stages of clinical development [85, 86]. A phase I trial of BMS-936558, a humanized IgG4 anti-PD-1 mAb, was conducted in patients with advanced solid tumors including NSCLC, renal carcinoma and melanoma. There were 31 responses; 20 of these were durable, lasting greater than one year. Immune adverse events, including pneumonitis, vitiligo, colitis, hepatitis, did not limit treatment. Of note, objective responses correlated with PD-L1 expression on tumor. Similarly, a large phase I study of a humanized IgG4 anti-PD-L1 mAb (BMS-936559), documented durable objective responses in 6-17% of patients with advanced solid tumors. Given the expression of PD-L1 in the majority of HPV(-) and HPV(+) HNSCC, these therapeutic antibodies are of particular interest in HNSCC - as monotherapy, or as adjuncts to conventional therapies including cetuximab.

## Therapeutic cancer vaccines

Enthusiasm for the development of head and neck tumor vaccines is motivated by the observation of nascent CTL responses against unique tumor antigens (TAs); the existence of this thwarted immune response implies the potential to harness and amplify adaptive immunity. Fundamentally, the goal of therapeutic cancer vaccination is inculcation of a persistent, TA-specific T cell response which kills tumor cells - abating tumor progression or even resulting in cure. In general, an effective vaccine will require successful TA presentation by professional APCs and a consequent TA-specific CTL response. Vaccines may target two forms of TAs: tumor-specific antigens (TSAs), or tumor-associated antigens (TAAs) [87]. TSAs are oncoproteins unique to the tumor not occurring in normal host cells (e.g. mutated p53 protein or the E6/E7 HPV oncoproteins). Targeting TSAs may be advantageous as these proteins are often central to tumorigenesis and their specificity would avoid auto-immune sequelae for normal tissue. However, a TSA-targeting vaccine may be applicable to only a small minority, whose tumor bears the candidate somatic mutation. This can be particularly prohibitive when the target is a tumor suppressor gene inactivated by a variety of point mutations, frameshifts or deletions - as is the case for p53 mutation, the most common genetic mutation in HNSCC [88]. TAAs are proteins over-expressed in tumor cells; however, they are also expressed in normal tissues (e.g. wild type EGFR). While TAA over-expression is prevalent in tumors with a common histology, making them a broadly applicable target, they are limited by weak immunogenicity and self-tolerance.

Ultimately, cancer vaccines must deliver antigenic peptides to professional APCs for presentation in association with MHC to the cognate CTLs. Various vaccination methods exist in HNSCC, each with their own particular advantages and drawbacks: 1) protein-based or peptide vaccines, consisting of pre-assembled proteins; 2) DNA vaccines, consisting of recombinant, TA-encoding DNA in a plasmid backbone; and 3) recombinant vector-based vaccines, where a viral, bacterial or yeast vector is loaded with recombinant DNA encoding the TA of interest. In peptide vaccines, for example HPV oncoprotein peptide vaccines, oncogenic activity must be inactivated while maintaining sufficient peptide length to stimulate CTL response. Advantages to this approach include ease of production and the ability to target TSA, whereas disadvantages include host proteolysis, weak immunogenicity, HLA restriction and poor long-lasting immunity [89]. DNA vaccines are more stable than peptides, however DNA uptake by APC associated with effective antigen expression is limited. Delivery methods, such as by electroporation or gene gun, can enhance uptake and immunogenicity [90]. Vector-based vaccines may overcome the poor antigenicity of naked DNA vaccines, due to a cross-over effect from the robust inflammatory response against vector antigens.

HPV is an ideal vaccine target, due to the expression of non-host TSAs and constitutive expression of these viral oncoproteins to maintain the transformed state. Proof-of-principle has been demonstrated by the successful development of HPV prevention vaccines, Cervarix^®^ and Gardasil^®^. While these marketed vaccines prevent anogenital HPV infection, their impact on the natural history of oral HPV is still unknown. Regardless, the capsid antibodies triggered by these L1 peptide vaccines are useful only for primary prevention; humoral blockade of the viral entry step is not relevant for established, HPV-transformed malignancies. Therapeutic vaccines for HPV-related cancers are of substantial interest in HNSCC. Five promising vaccination strategies have entered clinical development in HPV-induced neoplasia including two peptide vaccines, a detoxified E7 DNA vaccine, and two vector vaccines: 1) The HPV 16 E6 and E7 long peptide vaccine with incomplete freund's adjuvant was studied in 20 women with HPV-16 associated vulvar intraepithelial neoplasia. All patients had vaccine-induced CTL responses; 15 out of 19 patients had clinical responses [91]. 2) In a phase I study of a Trojan peptide vaccine containing HLA-I and HLA-II restricted Melanoma Antigen E (MAGE-A3) and HPV-16 derived peptides, immunogenicity was documented in 4 out of 5 patients with advanced HNSCC, however none exhibited an objective response [92]. 3) The HPV pNGVL4a-CRT/E7 (Detox) DNA vaccine contains the HPV 16 E7 gene engineered to disrupt the retinoblastoma binding site, thereby abrogating oncogenicity, embedded in the pNGVL-4a plasmid backbone [93]. This vaccine is under phase I study in patients with HPV-associated HNSCC following definitive multimodality therapy (NCT01493154). 4) TG4001, a modified vaccinia virus expressing the HPV-16 oncoproteins E6 and E7 as well as human interleukin- 2 (IL-2), has been studied in 21 patients with cervical intraepithelial neoplasia (CIN). HPV-16 clearance was associated with cytologic regression in 7/10 clinical responders. Additionally, 7/8 patients cleared HPV infection without conization and had no residual suspicion of CIN2/3 [94]. 5) The Lm-LLO-E7 vaccine harnesses a live-attenuated Listeria monocytogenes bacterium engineered to secrete the HPV-16 E7 antigen fused to listeriolysin O, the virulence factor permitting cytosolic replication in APCs [95]. This vaccine was evaluated for safety in 15 patients with advanced cervical carcinoma [96]. Dose-limiting toxicities consisted of pyrexia and diastolic hypotension; assessment of CTL response was technically limited. This vaccine is current under phase I investigation in patients with HPV-associated HNSCC with no evidence of disease after completion of standard therapy (NCT 01598792).

In HPV(-) HNSCCs, over-expressed wild type (wt) TAAs, such as p53, are potential vaccine targets. Although p53 mutation is the most commonly identified mutation in HPV(-) HNSCCs, most mutations result in the accumulation of p53; non-mutated portions of the protein are susceptible to degradation into wt peptide sequences appropriate for immune presentation. A phase I trial (NCT00404339) examining p53 multiple-epitope/dendritic cell vaccine in HNSCC patients was reported in 2009. Following definitive therapy, patients with locally advanced HNSCC were vaccinated with wt p53 sequences pre-loaded onto autologous dendritic cells. At 15-month follow up 11/16 patients were alive without disease. Analysis of immunogenicity indicated p53-specific CTLs in 5/16 patients [97].

## Current management of HPV-induced HNSCCs

Despite treatment intensification for patients with HNSCC, including altered radiation fractionation and the addition of chemotherapy to radiation, physicians and patients still face the significant challenge of recurrent or second tumors arising within or in close proximity to previously irradiated tissues. Locoregional recurrences develop in ~20% of patients treated with definitive chemoradiation for larynx preservation [98] or with post-operative chemoradiation for high-risk HNSCC [99, 100] and 17-33% of patients treated with definitive chemoradiation for locally advanced un-resectable disease [101, 102]. Locally recurrent tumors may arise from residual neoplastic cells that survive initial treatment, perhaps because of biological parameters that confer radio-resistance [103] or insufficiencies in initial treatment parameters such as radiation dose, volume, fractionation and treatment duration. Second cancers may arise from underlying field cancerization [104], as a radiation-induced malignancy, or as a de novo process and may be indistinguishable from a local recurrence of the primary tumor [105, 106].

Patients with recurrent HNSCC after prior radiation are a heterogeneous group. Differences in the location and extent of recurrent tumor, initial radiation treatment parameters, elapsed time since prior treatment, and extent of normal tissue sequelae, as well as relatively sparse data on acute and late normal tissue recovery from prior treatment and tolerance to re-irradiation [107], pose a significant challenge to the formulation of widely applicable schemata for re-irradiation. The optimal treatment volume for re-irradiation is uncertain. In an effort to limit the toxicity of re-treatment, many reported experiences with re-irradiation have targeted the recurrent gross disease with limited margin and not added elective nodal re-irradiation.

Despite the absence of evidence from randomized, controlled trials to support a de-escalation of treatment intensity in HPV(+) oropharyngeal carcinomas, some investigators argue that intensive concomitant chemoradiation regimens may represent overtreatment [108, 109]. Actually, an aggressive multimodality strategy, which may result in high rates of acute and long-term severe toxicity, would be not appropriate for HPV(+) patients who are younger and have prolonged survival. In this context, most efforts are targeted toward de-escalation of treatment intensity in HPV(+) SCCs with the intent to reduce toxicity and thereby improve the long-term quality of life, while maintaining efficacy. Recommended treatment de-escalation can be achieved by reducing the total dose of radiotherapy in a concurrent chemoradiotherapy setting, by using radiotherapy and EGFR inhibitors instead of cis-platinum based chemoradiotherapy or radiotherapy alone instead of chemoradiotherapy, and primary surgery +/− de-intensified adjuvant treatment instead of up-front chemoradiotherapy.

Aside from the Phase II Eastern Cooperative Oncology Group (ECOG) study and the Phase III Quarterback Trial, there are no active trials addressing radiotherapy dose. The Phase II ECOG study [110] confirmed the improved survival outcomes for patients with HPV(+) HNSCC observed in retrospective survival analyses. Also, these improved survival outcomes were consistent with an increased sensitivity of these cancers to chemotherapy and chemoradiation.

Nevertheless, a de-escalation strategy is not without concerns. A phase III non-inferiority trial for HPV(+) patients is considered difficult to conduct due to the large number of patients required [111]. Moreover, although HPV positivity results in a platform-independent survival benefit, the absolute superiority of any given platform is not yet known. Currently, several randomized controlled clinical trials specifically designed to test the efficacy of a de-intensification strategy in HPV(+) patients are on-going. These de-escalation protocols are mainly based on decreasing the intensity of the radiotherapy or on substituting cis-platinum with cetuximab in concurrent chemotherapy regimens. Treatment de-escalation strategies carry a risk of negatively impacting the overall favorable outcome of the patients. Several investigators sustain that the more favorable prognosis in HPV(+) SCCs may be attributable to better compliance to chemoradiotherapy strategies. Furthermore, emerging data suggest that cetuximab-radiotherapy may not be the preferred therapy in patients with HPV(+) cancers [112]. Very recently, a single-institutional experience with definitive radiation alone for HPV(+) HNSCC confirmed the inherent radio-sensitivity of these tumors [113]. Overall, there is insufficient evidence to treat HPV(+) SCCs with a de-intensified treatment strategy. This option should be restricted to controlled clinical trial settings with closely monitored safety assessments. Undoubtedly, it seems reasonable to exclude non-smoker patients with HPV(+) SCC from clinical trials using intensification of standard treatment. To date, the treatment of patients with HPV(+) OSCC should not be different from standard treatment of patients with HPV(-) tumors. It should be based on stage of disease and the general conditions of the patient, maximizing the probability to treat early stage SCCs with a single modality therapy [114].

Patients with head and neck cancer experience significant changes in their quality of life (qol) associated with disease and the adverse effects of treatment. Frequent problems the patients have to face are usually difficulties with speech, respiration and eating, apart from the psychological impact of loss of function and physical mutilation. These concerns associated with traditional trans-cervical surgical exposure approaches were principal in the clinical development of non-surgical treatment approaches based on fractionated radiotherapy. Over the past 30 years, multiple randomized trials have now established that treatment intensification with the addition of concurrent chemotherapy and altered radiotherapy fractionation schedules [115] can improve locoregional disease control rates and survival. It is also clear that these treatment intensification approaches can also contribute to an increased risk of late swallowing complications, raising concerns that such treatment approaches are also compromising qol and function [116]. As new treatment approaches are developed, prospective qol and function assessment are integral to the assessment in addition to traditional oncologic outcome measures. Development of the trans-oral robotic surgery (TORS) has greatly facilitated the trans-oral surgical approach for oropharyngeal carcinomas, evading many technical restraints [117].

Nevertheless, the optimal treatment for HPV(+) HNSCC patients remains uncertain. HPV(+) cancers appear more sensitive to chemoradiation as patients with low risk HPV(+) oropharyngeal cancers have almost double the overall survival as patients high risk HPV(-) cancers. This benefit in HPV(+) patients results from improved locoregional control rather than decreased distant metastasis. Since concurrent chemoradiation at least doubles the rate of acute and long term toxicities, less intense treatment regimens maximizing cure and decreasing toxicities are being investigated. To de-intensify the current standard of care would require reducing the current radiation dose and/or the chemotherapy regimens.

To this end, the ECOG Phase II trial (E1308) addressed these questions by testing the efficacy of decreasing the radiation dose. Patients achieving a complete response to induction chemotherapy were treated with lower dose radiation and cetuximab. The fact that cetuximab is an antibody targeting the cancer cell membrane and is thus associated with lower toxicity, renders radiation with cetuximab or bioradiotherapy distinct from chemoradiotherapy. Nevertheless, it remains unclear whether bioradiotherapy provides as good locoregional control as chemoradiotherapy. A retrospective analysis showed that bioradiotherapy may not be as effective as chemoradiation, especially in patients with HPV(+) cancers [118]. Similarly, a recent trial suggested that bioradiotherapy has more local failures than chemoradiotherapy in patients with laryngeal cancers [119]. Nevertheless, the ECOG trial is a major advance towards treatment de-intensification even though there was no direct comparison between bioradiotherapy and chemoradiotherapy.
